# The EnvZ-OmpR Two-Component Signaling System Is Inactivated in a Mutant Devoid of Osmoregulated Periplasmic Glucans in *Dickeya dadantii*

**DOI:** 10.3389/fmicb.2018.02459

**Published:** 2018-10-30

**Authors:** Marine Caby, Sébastien Bontemps-Gallo, Peggy Gruau, Brigitte Delrue, Edwige Madec, Jean-Marie Lacroix

**Affiliations:** ^1^Unité de Glycobiologie Structurale et Fonctionnelle, UMR CNRS 8576, Université des Sciences et Technologies de Lille, Université de Lille, Lille, France; ^2^Université de Lille, Lille, France

**Keywords:** EnvZ/OmpR, osmoregulated periplasmic glucans, osmotic stress, plant pathogen, *D. dadantii*

## Abstract

Osmoregulated periplasmic glucans (OPGs) are general constituents of alpha-, beta-, and gamma-Proteobacteria. This polymer of glucose is required for full virulence of many pathogens including *Dickeya dadantii (D. dadantii)*. The phytopathogenic enterobacterium *D. dadantii* causes soft-rot disease in a wide range of plants. An OPG-defective mutant is impaired in environment sensing. We previously demonstrated that (i) fluctuation of OPG concentration controlled the activation level of the RcsCDB system, and (ii) RcsCDB along with EnvZ/OmpR controlled the mechanism of OPG succinylation. These previous data lead us to explore whether OPGs are required for other two-component systems. In this study, we demonstrate that inactivation of the EnvZ/OmpR system in an OPG-defective mutant restores full synthesis of pectinase but only partial virulence. Unlike for the RcsCDB system, the EnvZ-OmpR system is not controlled by OPG concentration but requires OPGs for proper activation.

## Introduction

Osmoregulated periplasmic glucans (OPGs), β-D-glucans oligosaccharides, are major envelope components found in the periplasm of almost all proteobacteria. Their concentration increases as the osmolarity of the medium decreases (Kennedy, 1996; Bohin and Lacroix, 2006; Bontemps-Gallo et al., 2017). In enterobacteria, the gene products of the *opgGH* operon synthesize the OPG glucose backbone, which is composed of 5–12 glucose units joined by β,1-2 linkages and branched by β,1-6 linkages. The *opgG* and *opgH* mutant strains are completely devoid of OPGs (Bontemps-Gallo et al., 2017). These glucans are well described as virulence factors of animal and plant pathogens including *Dickeya dadantii (D. dadantii)* (Bontemps-Gallo and Lacroix, 2015).

*D. dadantii*, the agent of soft rot disease, is directly responsible for 5 to 25% of potato crop loss in Europe and Israel (Toth et al., 2011). This phytopathogen is listed as an A2 quarantine organism by the European and Mediterranean Plant Protection Organization(EPPO, 1982, 1988, 1990). Maceration is the result of the synthesis and secretion of plant cell wall-degrading enzymes (PCWDEs), in particular, pectinases (Collmer and Keen, 1986). However, additional factors, such as motility, are required for full virulence (Charkowski et al., 2012; Reverchon and Nasser, 2013; Leonard et al., 2017). During infection, *D. dadantii* must overcome several stressors including osmotic stress. Previous studies suggest that bacteria encounter hypoosmotic stress at the early stage of infection and hyperosmotic stress later due to plant maceration (Reverchon and Nasser, 2013; Jiang et al., 2016; Reverchon et al., 2016).

In our model, OPG concentration dramatically increases during the first hour of infection (Bontemps-Gallo et al., 2013). Mutants devoid of OPGs show a pleiotropic phenotype including a loss of motility, decreased synthesis and secretion of PCWDEs, increased synthesis of exopolysaccharide, induction of a general stress response, and complete loss of virulence on potato tubers or chicory leaves (Page et al., 2001; Bouchart et al., 2007). These phenotypes suggest that strains lacking OPGs are impaired in the sensing of their environment. Previously, our laboratory demonstrated a strong relationship between OPGs and the RcsCDB two-component system.

Two-component systems are key regulators of gene expression plasticity in response to environmental changes. Under stimuli, often unknown, a transmembrane sensor histidine kinase (HK) autophosphorylates on a histidine residue. This phosphate group is subsequently transferred to an aspartate residue on a cognate cytoplasmic response regulator (RR), which in turn regulates the expression of a set of target genes (Hoch, 2000; Groisman, 2016).

Inactivation of the RcsCDB system in an OPG-defective mutant restores several of the *D. dadantii* wild-type phenotypes (motility, mucoidy, and virulence) (Bouchart et al., 2010), indicating that OPGs are involved in the perception of environmental changes. We have also shown that RcsCDB and OPG are tightly connected: (i) fluctuation of OPG concentration controls the activation level of the RcsCDB system (Bontemps-Gallo et al., 2013), and (ii) RcsCDB, along with the two-component system EnvZ/OmpR, controls the mechanism of OPG succinylation (Bontemps-Gallo et al., 2016). These facts lead us to question whether the link between OPGs and the RcsCDB system is a unique feature.

Thirty years ago, Fiedler and Rotering (1988) isolated revertants in OPG-defective mutants of *E. coli*. The mutation was localized to the *ompB* locus now known as the *envZ*-*ompR* operon. EnvZ-OmpR, the paradigm of two-component systems, regulates the balance between OmpF (large pore diameter) and OmpC (small pore diameter) to control the diffusion rate of nutrients (Cowan et al., 1992; Forst and Roberts, 1994; Egger et al., 1997; Castillo-Keller et al., 2006; Barbieri et al., 2013). This system is also known to control motility in several bacteria (Barker et al., 2004; Clemmer and Rather, 2007; Raczkowska et al., 2011; Lee and Park, 2013; Li et al., 2014; Tipton and Rather, 2016; Pruss, 2017) and is required for full virulence in *Yersinia pestis* (Gao et al., 2011; Reboul et al., 2014). In *D. dadantii*, the EnvZ/OmpR system regulates *ompF* expression (no *ompC* homolog is present) as well as *kdgN*, which is required for transport of oligosaccharides arising from pectin degradation during plant infection (Condemine and Ghazi, 2007). Recently, in a global *in vitro* transcriptomic analysis of various stressors encountered during the infectious process, Jiang et al. (2016) showed that the EnvZ-OmpR system was up-regulated during osmotic stress.

In this study, we demonstrate that EnvZ-OmpR system is not involved in virulence. Instead, inactivation of *envZ* or *ompR* in an OPG-defective mutant restores full synthesis of pectinase and partial virulence. We also show that EnvZ-OmpR is involved in regulation of motility. Finally, we demonstrate that *ompF* and *kdgN* are osmoregulated by EnvZ-OmpR and are required for proper regulation of OPGs.

## Materials and Methods

### Bacterial Strains, Media, and Growth Conditions

Bacterial strains are described in Table 1. Bacteria were grown at 30°C in lysogeny broth (LB) (Bertani, 2004), or in minimal medium M63 glycerol [15 mM (NH_4_)_2_SO_4_, 1.8 μM FeSO_4_, 1 mM MgSO_4_, and 100 mM K_2_PHO_4_] supplemented with 0.2% glycerol as a carbon source (Miller, 1992). Solid media were obtained by adding agar at 15 g.L^-1^. Motility tests were performed on LB plates containing agar at 4 g.L^-1^.

**Table 1:** Strains used in the study.

Strain	Relevant Genotype and/or phenotype^a^	Source or reference
EC3937	Wild-type	Laboratory collection
NFB3723	*opgG*::Cml	Bontemps-Gallo et al., 2013
NFB3835	*opgG*::Cml miniTn5 P_BAD_*-opgGH*-Spe	Bontemps-Gallo et al., 2013
NFB7422	*ompR*::Gm	Bontemps-Gallo et al., 2016
NFB7423	*ompR*::Gm *opgG*::Cml	This study
NFB7440	*ompR*::Gm *opgG*::Cml miniTn5 P_BAD_*-opgGH*-Spe	This study
NFB7515	*cpxA*::Gm	Bontemps-Gallo et al., 2015
NFB7521	*envZ*::Gm	Bontemps-Gallo et al., 2016
NFB7524	*envZ*::Gm *opgG*::Cml	This study
NFB7532	*cpxR*::Gm	Bontemps-Gallo et al., 2015
NFB7534	*cpxR opgG*::Cml	This study
NFB7632	*cpxA*::Gm *opgG*::Cml	This study
NFB7731	*envZ*::Gm *opgG*::Cml miniTn5 P_BAD_*-opgGH*-Spe	This study


*^a^Cml, chloramphenicol resistance; Gm, gentamicin resistance; Spe, spectinomycin resistance. P_BAD_-opgGH fusion is carried by a mini-Tn5.*

Osmolarity (mOsM) was measured with a vapor pressure osmometer (Advanced Instruments, United States). M63 osmolarity was 330 mOsM. Osmolarity was decreased by diluting twofold M63 with H_2_O to 170 mOsM. Addition of 0.1 and 0.2 M NaCl increased the osmolarity to 500 and 700 mOsM, respectively. Glycerol was added after dilution with water or addition of NaCl.

The solid media used to test the pectinase [M63 supplemented with 0.4% polygalacturonate (PGA) and 0.2% glycerol], cellulases [M63 supplemented with 0.2% carboxymethylcellulose (CMC), 0.2% glycerol, and 7 mM MgSO_4_], and proteases (LB complemented with 1% of Fat milk) activities have been described previously (Page et al., 2001).

Antibiotics were used at following concentrations: spectinomycin, 2.5 μg.mL^-1^; chloramphenicol, 12.5 μg.mL^-1^; and gentamycin, 2 μg.mL^-1^.

### Transduction, Conjugation, and Transformation

Construction of strains was performed by transferring genes from one strain of *D. dadantii* to another by generalized transduction with phage ΦEC2, as described previously (Resibois et al., 1984). Plasmids were introduced in *D. dadantii* by conjugation or electroporation.

### Expression Analysis

Bacteria were grown until the exponential phase at various osmolarities. RNAs were extracted using Nucleospin RNA Plus Kit (Macherey Nagel) following the manufacturer’s instructions. RNAs were treated with DNase I (BioLabs). RNA qualities were checked by gel and nanodrop.

cDNAs were retrotranscribed using the Superscript IV First-Strand Synthesis (Invitrogen) according to the manufacturer’s instructions.

qPCR was performed using SYBR method as described previously by Hommais et al. (2011). Primers used are listed in Table 2. Further, *ipxC*, an UDP-N-acetylglucosamine deacetylase, was used as a reference gene (Hommais et al., 2011).

**Table 2:** qPCR primers.

Primer	Sequence	Efficiency	Reference
ompF-F	CGT AAC TCT GGT GTT GCT ACT T	1.843	This study
ompF-R	AGT CGC TAT GTG CTG ATT GG		
kdgN-F	CCT GCG TTA TCG TCC TTT CTA C	1.428	This study
kdgN-R	CAG CAC GCT GGT AAT GGT ATA G		
ompR-F	GCT CGA TTG ATG TGC AGA TTT C	1.904	This study
ompR-R	ACA AAG ACG TAG CCC AAC C		
envZ-F	CTG GCG GAG TCG ATC AAT AA	1.652	This study
envZ-R	GCC ACT TCC ATC TGC ATT TC		
spy-F	CGG AAG GCG TAG TCA ATC AA	1.943	This study
spy-R	TTT CTG TTC CGG CGT CAA		
degP-F	CCA GAT TGT CGA ATA CGG AGA G	1.733	This study
degP-R	GCA TCC ACT TTC ATG GCT TTA G		
opgG-F	CCG GAA CAG GCT TAT GTG AT	1.774	This study
opgG-R	AAT CGA CCA GGA ATG CAG TAG		
opgH-F	GGA ACT GGC GAT AGC TTT GT	1.547	This study
opgH-R	CCA CTC CGC CGT ATG ATT TAG		
flhD-F	TCG GTT GGG TAT CAA TGA AGA A	1.815	This study
flhD-R	TCA CTG AAG CGG AAA TGA CAT A		
fliC-F	CAC GGC TCA TGT TGG ATA CT	1.676	This study
fliC-R	CA TTG ACA ACC TGA GCA ACA C		
ipxC-F	AAA TCC GTG CGT GAT ACC AT	1.862	Hommais et al., 2011
ipxC-R	CAT CCA GCA GCA GGT AGA CA		


### Phenotypic Evaluation

A total of 10^7^ bacteria in 5 μL were spotted onto pectinase (PGA), cellulase (CMC), protease or motility plates. After 48 h incubation, the PGA plates were flooded with a 10% copper acetate solution, which forms a blue complex with the PGA. Diameters of the clear haloes around the colony were measured as an indication of pectinase production. After 48 h incubation, CMC plates were flooded with a 1 mg/ml red Congo solution and washed several times with 1 M NaCl, allowing formation of a red complex with the CMC. Diameters of the clear haloes around the colony were measured as an indication of cellulase production. After 48 h of incubation, the abilities of the strain to degrade milk protein were observed. Swim diameters were measured after 48 h of incubation.

### Pathogenicity Test

Potato tubers and chicory leaves were inoculated as previously described (Page et al., 2001). Bacteria from an overnight culture in LB medium were recovered by centrifugation and diluted in water. For potato tubers, sterile pipette tips containing a bacterial suspension of 10^7^ cells in 5 μL were inserted into the tuber (Amandine variety). After 72 h of incubation in a dew chamber, the tubers were sliced vertically through the inoculation point, and the weight of the maceration was measured. For chicory leaves, the leaves were wounded prior to inoculation of 10^7^ bacteria and incubated in a dew chamber at 30°C until 48 h.

### Transmission Electron Microscopy

Samples were analyzed by the Bio Imaging Center of University of Lille (France). Wild-type and *opgG* strains were grown until the mid-log phase. Cells were spun for 5 min at 7,000 × *g* at 4°C. Bacteria were fixed with 3.125% glutaraldehyde, washed in 0.1 M phosphate buffer pH 7.4, and postfixed with 1% OsO_4_. The samples were dehydrated with graded acetone series, embedded in EMBED resin, and air dried at 60°C. Thin and ultrathin sections were prepared using an ultramicrotome (Reichert OM U3 or LKB Ultrotome III 8800) and stained with uranyl acetate. Microscopy was performed with a Hitachi H600 microscope at 75 keV electron energy. The periplasm length was measured using ImageJ software.

### Statistical Analysis

For statistical analyses, Graph-prism 6 software was used to analyze the data using one-way ANOVA.

## Results

### Characterization of *envZ* and *ompR* Deletion in Wild-Type and *opgG* Background

**FIGURE 1:**
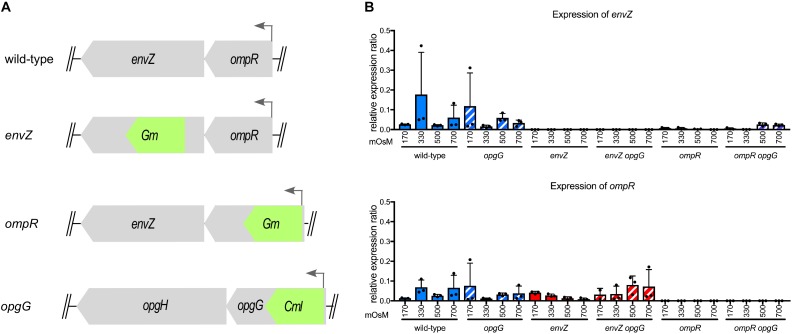
Characterization of the *envZ* and *ompR* deletion in wild-type and *opgG* background. **(A)** Schematic of the *envZ*-*ompR* locus in the wild-type strain and genetic organization of the mutant strains. **(B)** Expression of *envZ* and *ompR* was analyzed by qPCR. Bacteria were grown at 170, 330, 500, and 700 mOsM. Relative gene expression was calculated using *ipxC* as a reference (Hommais et al., 2011). Data represent mean ± standard deviation of three independent experiments.

To determine whether the EnvZ-OmpR system interacts with OPG, we inactivated *envZ* or *ompR* in wild-type and *opgG* mutant backgrounds (Figure 1A). We then looked at *envZ* and *ompR* expression at various osmolarities (Figure 1B). As expected, the expression of both genes was low in the wild-type strain and not affected by osmolarity. In an *opgG* mutant, the expression level was similar to that observed in the wild-type strain. No expression of *envZ* or *ompR* was observed in their respective mutant strain. Interestingly, in the *ompR* background, a low but measurable expression of *envZ* was observed. Based on the locus organization, we would expect the *ompR* mutation to be polar. Expression of *envZ* in an *ompR* deletion background suggests the presence of a secondary promoter.

### Inactivation of *envZ* or *ompR* Restores the Synthesis of Pectinase in an OPG-Defective Strain

**FIGURE 2:**
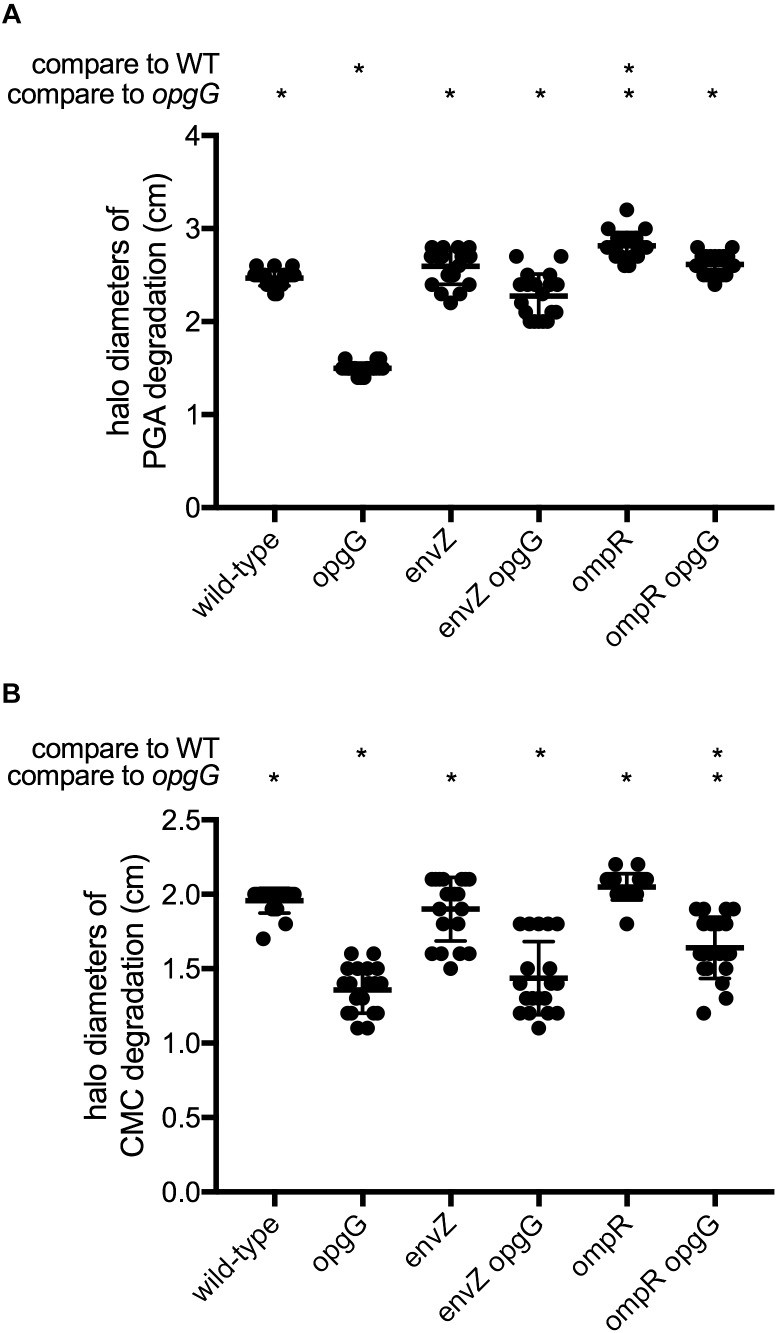
Pectinase **(A)** and cellulase **(B)** activities. Exoenzyme activities were estimated on plates by the measurement of halo diameters, expressed in cm of substrate degradation. Data represent mean ± standard deviation of 20 independent experiments. An asterisk indicates a significant difference with *p* < 0.0001.

Strains devoid of OPGs are impaired in their ability to synthesize virulence factors, leading to a total loss of virulence. We first assayed plant cell-degrading enzyme activity (Figure 2 and Supplementary Figure 1), which is required for full virulence. Pectinase production and secretion were evaluated on a minimal medium containing polygalacturonate, a substrate for pectinase, and after 48 h of incubation, haloes of degradation were measured (Figure 2A). As expected, the *opgG* mutant showed a 40% decrease in pectinase production compared to the wild-type. While inactivation of *envZ* or *ompR* did not decrease the synthesis of pectinases, *envZ opgG* and *ompR opgG* double mutants showed full restoration of pectinase production to levels similar to the wild-type.

Cellulase production and secretion were evaluated on a minimal medium containing carboxymethylcellulose, the substrate for cellulase, and haloes of degradation were measured after 48 h of incubation (Figure 2B). As previously shown, *opgG* inactivation decreased the production of cellulase by 30% (Page et al., 2001). The *envZ* or *ompR* null mutants exhibited similar cellulase levels to the wild-type. The *envZ opgG* and *ompR opgG* double mutants displayed a reduction in cellulase production similar to the *opgG* strain.

We also assayed for the production of protease on plates containing 1% milk fat (Table 3). The ability of each strain to degrade milk protein was evaluated after 48 h. No restoration of protease activity was observed in any of the double-mutant strains.

**Table 3:** Protease activity.

Strain	
Wild-type	+
*opgG*	-
*envZ*	+
*envZ opgG*	-
*ompR*	+
*ompR opgG*	-


*Protease activities were observed on plates by the presence of a clear halo and marked as “+.” Data represent observations from three independent experiments.*

Taken together, our data show that EnvZ-OmpR is not involved in the regulation of PCWDEs. However, disruption of either *envZ* or *ompR* is enough to restore full pectinase production in an OPG-defective strain, but not cellulase or protease synthesis.

### The EnvZ-OmpR System Is Involved in Motility Regulation

**FIGURE 3:**
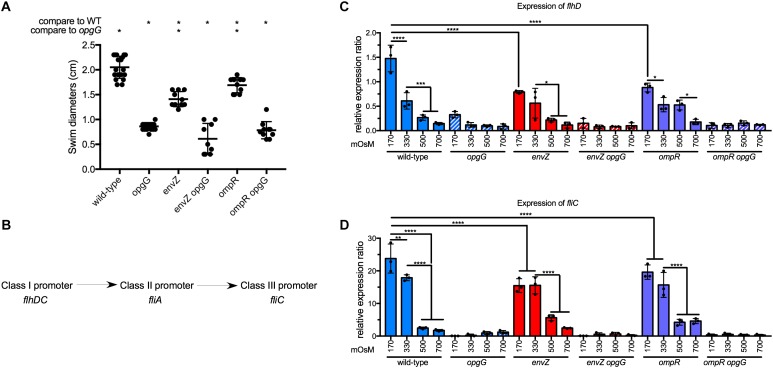
Effect of EnvZ-OmpR on motility in wild-type and *opgG* background. **(A)** Motility of wild-type, *opgG, envZ, envZ opgG, ompR*, and *ompR opgG* strains. Motility was measured in M63 semisolid plates. Swim diameters were measured after 48 h of incubation at 30°C. **(B)** Schematic of the regulatory cascade of motility. FlhDC, a master regulator and a class I promoter, modulates gene expression with a class II promoter (e.g., *fliA*). In return, the products of those genes regulate the genes with a class III promoter (e.g., *fliC*). **(C,D)** Expression of **(C)** *flhD* and **(D)** *fliC* in wild-type, *opgG, envZ, envZ opgG, ompR*, and *ompR opgG* strains. Bacteria were grown at 170, 330, 500, and 700 mOsM. The expression of **(C)** *flhD*, **(D)** *fliC* was analyzed by qPCR. Relative gene expression was calculated using *ipxC* as a reference (Hommais et al., 2011). Data represent mean ± standard deviation of 10 independent experiments. An asterisk indicates a significant difference with ^∗∗∗∗^*p* < 0.0001, ^∗∗∗^*p* < 0.001, ^∗∗^*p* < 0.01, and ^∗^*p* < 0.05.

Motility is known to be an important virulence factor (Reverchon and Nasser, 2013). Furthermore, by screening motility in OPG-defective mutants of *E. coli*, Fiedler and Rotering isolated revertants in the *envZ-ompR* operon (Fiedler and Rotering, 1988). To determine whether the disruption of *envZ-ompR* could restore the loss of motility in the *opgG* mutant, we assayed for motility by measuring swim diameters on 0.4% agar plates (Figure 3A and Supplementary Figure 1). As described previously, the *opgG* mutant showed a reduction in motility (one third of wild-type levels). Inactivation of *envZ* or *ompR* resulted in a 40% reduction in motility compared to the wild-type strain. However, the same mutation in the *opgG* background did not restore motility.

The regulatory cascade for motility is separated into three classes of promoters (Figure 3B). Under motility-inducing conditions, *flhDC*, the master regulator, is up-regulated to modulate the expression of genes under the control of a class II promoter. Finally, class II genes regulate genes with class III promoters (e.g., *fliC*, the flagellin). We then tested the effect of the EnvZ-OmpR system on the regulation of motility. In wild-type background, the expression of the master regulator *flhD*, and consequently *fliC*, decreased 10-fold from low (170 mOsM) to high (700 mOsM) osmolarity (Figures 3C,D). This data agrees with our previous observation of a twofold decrease in wild-type motility in the same osmolarity range (Bontemps-Gallo et al., 2013). Inactivation of *envZ* or *ompR* lead to a decrease in *flhD* expression but, save for 170 mOsM, this decrease was not statistically significant (Figure 3C). The *fliC* expression decreased 1.5-fold at 170 and 330 mOsM in the *envZ* and *ompR* mutants, respectively, compared to the wild-type (Figures 3C,D). Disruption of *opgG* resulted in low expression of both *flhD* and *fliC* regardless of the genetic background and osmolarity (Figures 3C,D). Our results show that EnvZ-OmpR is involved in the regulation of motility but not as a main regulator of this cascade. Inactivation of this system cannot rescue motility in the *opgG* background.

### Inactivation of EnvZ-OmpR Systems Partially Restores Virulence in an OPG-Defective Strain

**FIGURE 4:**
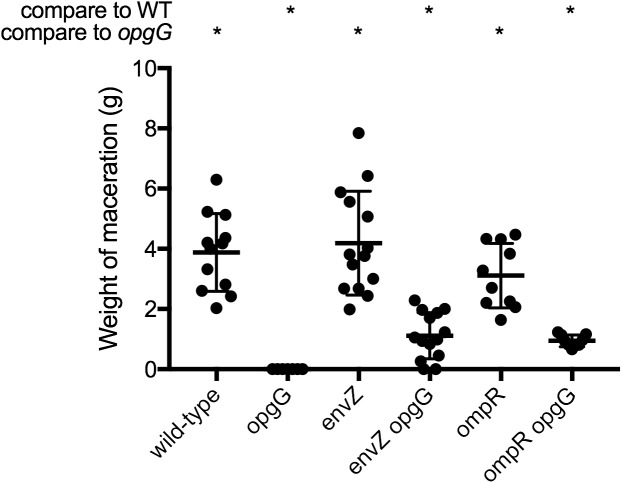
Weight of maceration on potato tubers for wild-type, *opgG, envZ, envZ opgG, ompR*, and *ompR opgG* strains. Bacteria were inoculated into holes on potato tubers. Maceration (g) was weighed after 72 h of incubation at 30°C. Data represent mean ± standard deviation of at least 10 independent experiments. An asterisk indicates a significant difference with *p* < 0.0001.

**FIGURE 5:**
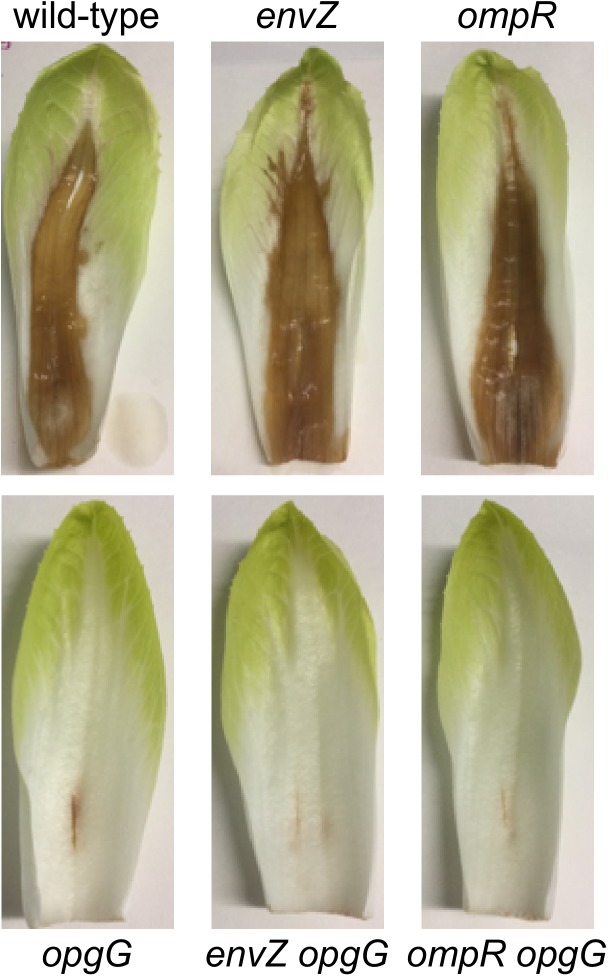
Pathogenicity of wild-type, *opgG, envZ, envZ opgG, ompR*, and *ompR opgG* strains on chicory leaves. Bacteria were inoculated into scarified chicory leaves. Disease symptoms were observed after 48 h of incubation at 30°C. The results presented are of one of the three independent experiments performed.

Previously, we demonstrated that restoration of pectinase production in an OPG-defective strain is enough to restore virulence in potato tubers but not in chicory leaves (Bontemps-Gallo et al., 2014). We observed that inactivation of the EnvZ-OmpR system in an *opgG* mutant lead to restoration of full pectinase synthesis (Figure 2A). We therefore determined whether inactivation of this system could restore virulence in both potato tubers (Figure 4 and Supplementary Figure 1) and chicory leaves (Figure 5). Following the inoculation of bacteria in both vegetables and incubation at 30°C, we analyzed the virulence levels. Inactivation of *envZ* or *ompR* in a wild-type background had no effect on the virulence levels regardless of the infection model used (Figures 4, 5). Interestingly, when the system was inactivated in an OPG-defective strain, macerations were observed in the tubers (Figure 4). However, the severity of disease was not as strong as for the wild-type strain (only a third of the average maceration weight of the wild-type). No restoration of virulence was observed for *envZ opgG* or *ompR opgG* double mutants in chicory leaves (Figure 5). Our data demonstrate that EnvZ-OmpR is not involved in virulence in *D. dadantii*. Furthermore, restoration of pectinase synthesis in the double mutants allows for maceration but only in potato tubers.

### *ompF* and *kdgN* Are Osmoregulated Through EnvZ-OmpR and Require OPG for Regulation

**FIGURE 6:**
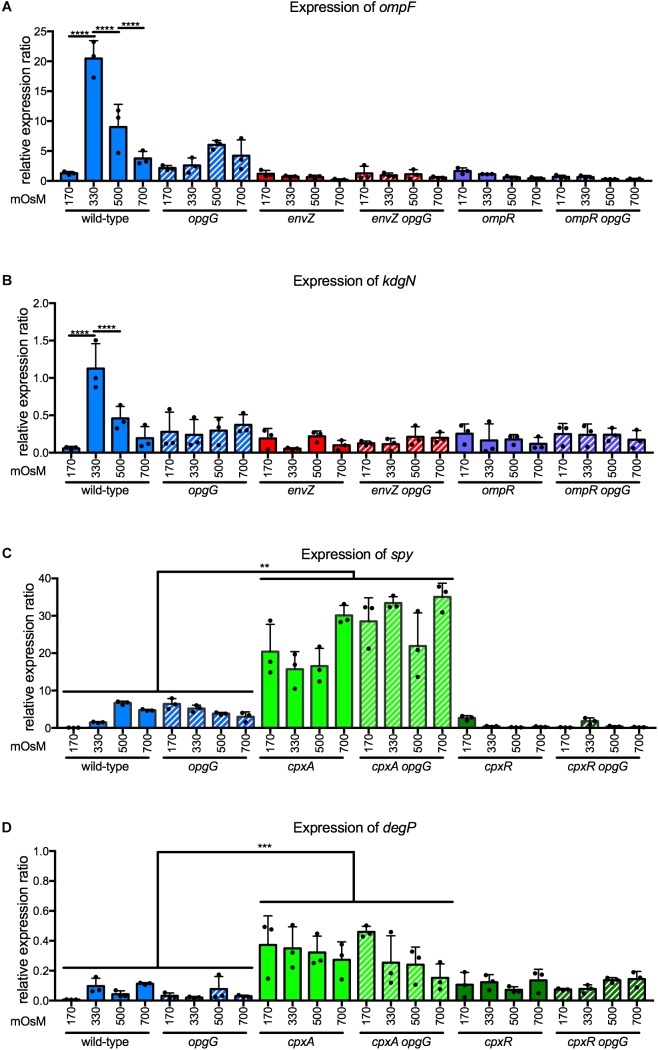
Expression of **(A)** *ompF* and **(B)** *kdgN* in wild-type, *opgG, envZ, envZ opgG, ompR*, and *ompR opgG* strains and of **(C)** *spy* and **(D)** *degP* in wild-type, *opgG, cpxA, cpxA opgG, cpxR*, and *cpxR opgG* strains at various osmolarities. Bacteria were grown at 170, 330, 500, and 700 mOsM. The expression of **(A)** *ompF*, **(B)** *kdgN* **(C)** *spy* and **(D)** *degP* were analyzed by qPCR. Relative gene expression was calculated using *ipxC* as a reference (Hommais et al., 2011). Data represent mean ± standard deviation of three independent experiments. An asterisk indicates a significant difference with ^∗∗∗∗^*p* < 0.0001, ^∗∗∗^*p* < 0.001, and ^∗∗^*p* < 0.01.

In *D. dadantii*, EnvZ-OmpR regulates at least two genes involved in transport – *ompF* and *kdgN* (Condemine and Ghazi, 2007). KdgN transports oligosaccharides arising from pectin-mediated degradation during plant infection. OmpF is a porin with a pore diameter of 1.12 nm that allows a non-specific import of hydrophilic metabolites of less than 600 Da. We analyzed the expression of these two genes at 170, 330, 500, and 700 mOsM in a wild-type background (Figures 6A,B). Expression increased 16-fold for *ompF* and 22-fold for *kdgN* between 170 and 330 mOsM. Subsequently, the expression decreased twofold for both genes between 330 and 500 mOsM, and twofold for *ompF* when osmolarity increased to 700 mOsM. In *envZ* or *ompR* single mutants, regulation was completely lost showing that *ompF* and *kdgN* are part of the regulon (Figures 6A,B). Both genes followed a classic bell curve observed for gene regulation by EnvZ-OmpR in *E. coli* (Lan and Igo, 1998). Interestingly, in the *opgG* mutant, regulation was completely lost (Figures 6A,B). At 170 mOsM, the expression level of *ompF* or *kdgN* in the OPG-defective strain was at a similar level to the wild-type, regardless of medium osmolarity. These data indicate that the EnvZ-OmpR system regulates the expression of *ompF* and *kdgN* in an OPG-dependent manner.

### OPGs Are Not Required for the Activation of the CpxAR Two-Component System

To show whether two-component system dysfunction is a general feature of bacteria lacking OPGs, we investigated the potential relationship between another two-component system and OPGs. Among the 32 two-component systems in *D. dadantii*, three systems are involved in sensing stress – RcsCDB, EnvZ-OmpR, and CpxAR. CpxAR is involved in the perception of envelope stress (Bontemps-Gallo et al., 2015). Inactivation of this system in an *opgG* background does not restore any phenotype (Bontemps Gallo, 2013). CpxAR regulates *spy*, encoding for a periplasmic chaperon, and *degP*, a periplasmic protease (Bontemps-Gallo et al., 2015). As previously observed, the expression of *spy* (Figure 6C) and *degP* (Figure 6D) were up-regulated in a *cpxA* background. In a *cpxR* mutant, *spy* was down-regulated and *degP* had an expression similar to wild-type (Figures 6C,D; Bontemps-Gallo et al., 2015). Disruption of *opgG* does not affect the regulation of *spy* or *degP* by the CpxAR system (Figures 6C,D). Taken together, our data shows that OPGs have a specific relationship with certain two-component systems.

### Periplasmic Size Is Maintained in an OPG-Defective Mutant

**FIGURE 7:**
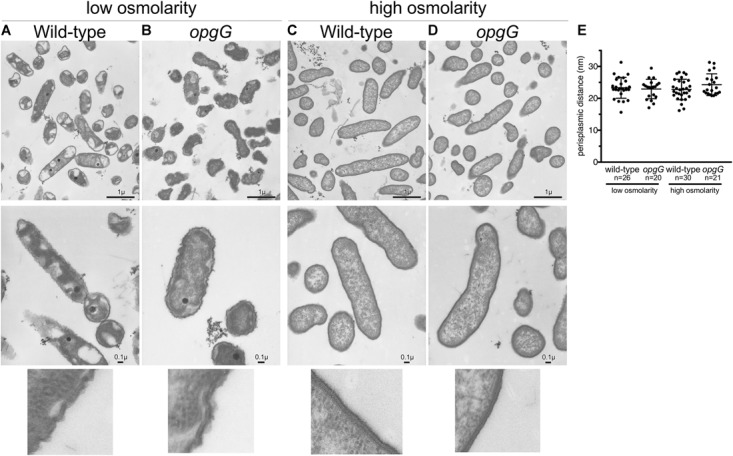
Transmission electron microscopy images of wild-type **(A,C)** and *opgG* mutant **(B,D)** at low osmolarity **(A,B)** and high osmolarity **(C,D)**. Images show similar architecture for both strains when grown in the same medium but differences when osmolarity is varied. **(E)** Periplasm size (nm) from TEM images.

Periplasmic size is subject to fluctuations during osmotic stress (Bohin and Lacroix, 2006) and loss of OPGs, and major periplasmic components representing up to 5% of the dry weight of a cell, could affect this size. Recently, Asmar et al. (2017) demonstrated that the activation of two-component systems also relies on the distance between the two membranes. To determine whether a change in periplasm width may be one of the consequences of a lack of EnvZ-OmpR system activation in the *opgG* mutant, we grew bacteria until mid-log phase in low and high osmolarities and analyzed the cell ultrastructure using transmission electron microscopy (Figure 7). At low osmolarity (Figures 7A,B), the cells exhibited an altered cytoplasmic content with small dense granules being observed. Since poly-phosphate granules, often accumulated by *D. dadantii*, typically appear white by TEM (Ogawa et al., 2000; Ayraud et al., 2005; Stumpf and Foster, 2005), we suspect that the black granules are filled with ferrous poly-phosphates (Lechaire et al., 2002). This cytoplasmic modification had no effect on the growth of *D. dadantii*. At high osmolarity (Figure 7), the cell displayed a classic rod-shaped form. Despite the strong structural difference observed for bacteria grown in low and high osmolarities, no significant difference was observed in the bacterial structure between the wild-type and the *opgG* mutant strains at any osmolarity. In addition, no relevant difference in periplasmic size was observed between the wild-type and the *opgG* mutant. Both strains displayed an equivalent periplasmic space: 23.99 nm ± 3.26 for wild-type and 22.92 nm ± 3.04 for the OPG-defective strain at low osmolarity and 22.23 nm ± 3.21 for wild-type and 24.28 nm ± 3.41 for the OPG-defective strain at high osmolarity (Figure 7E). This suggests that OPGs are not involved in the control of periplasmic size. These periplasmic space measurements are similar to those observed by Asmar et al. (2017) for the closely related *E. coli* Enterobacterium in LB medium (around 350 mOsM). Taken together, the gene expression experiments and the microscopy observations strongly suggest that EnvZ-OmpR requires OPGs in the periplasm to be able to sense the osmolarity, but this sensing is not based on periplasmic size.

### Increasing Concentrations of OPGs Do Not Affect the Level of EnvZ-OmpR System Activation

**FIGURE 8:**
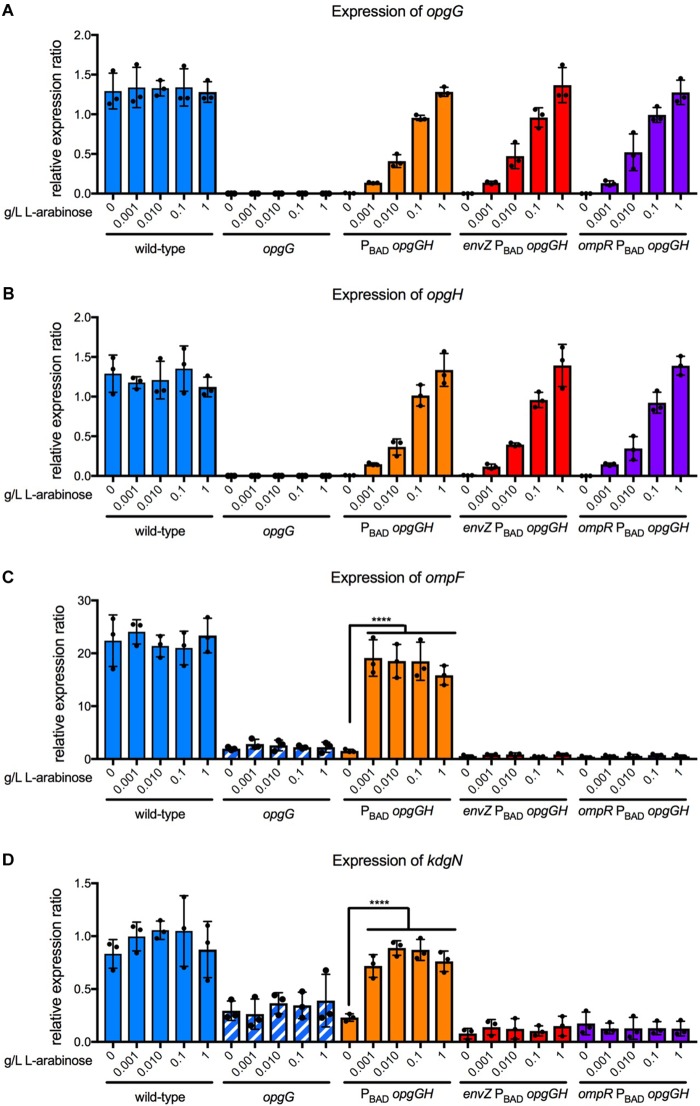
Effect of OPG concentration on expression of *opgG, opgH, ompF*, and *kdgN*. Bacteria were grown in M63 medium (330 mOsM) with increasing L-arabinose concentration ranging from 0 to 1 g/L. The expression of **(A)** *opgG*, **(B)** *opgH*, **(C)** *ompF*, and **(D)** *kdgN* was analyzed by qPCR. Relative gene expression was calculated using *ipxC* as a reference (Hommais et al., 2011). Data represent mean ± standard deviation of three independent experiments. An asterisk indicates a significant difference with ^∗∗∗∗^*p* < 0.0001, ^∗∗∗^*p* < 0.001, ^∗∗^*p* < 0.01, and ^∗^*p* < 0.05.

Previously, we demonstrated that the level of RcsCDB activation is controlled by the concentration of OPGs (Bontemps-Gallo et al., 2013). Therefore, we examined whether the concentration of OPGs could also modulate the level of EnvZ-OmpR activation (Figure 8). For this, we used a system in which the *opgGH* operon is under the control of the P_BAD_ promoter from *E. coli*. Control of L-arabinose concentration enables tight regulation of the *opgGH* operon (Guzman et al., 1995). We grew the P_BAD_-*opgGH, envZ* P_BAD_-*opgGH, ompR* P_BAD_-*opgGH*, as well as the wild-type and *opgG* strains, in a M63 medium at various L-arabinose concentrations ranging from 0 to 1 g/L. We first confirmed that the expression of the *opgG* and *opgH* genes increased in line with the increasing concentration of L-arabinose (Figures 8A,B). As shown previously, without L-arabinose, no OPG is detected. OPG concentration increased in accordance with the L-arabinose concentration, as described previously (Bontemps-Gallo et al., 2013). We then analyzed the expression of *ompF* and *kdgN* in the same strains under the same conditions (Figures 8C,D). Without L-arabinose, the expression of *ompF* and *kdgN* in the P_BAD_-*opgGH* strain was similar to that measured for the *opgG* mutant (Figures 6, 8C,D). In the presence of L-arabinose, regardless of the concentration, the expression of both genes was similar to the expression in the wild-type strain (Figures 6, 8C,D). Inactivation of either *envZ* or *ompR* in the P_BAD_-*opgGH* strain lead to a low expression level regardless of the presence of L-arabinose. Our data show that OPGs are required for the transmission of the sensing signal but they do not control the level of EnvZ-OmpR activation.

## Discussion

Since their first characterization in 1973 by E.P. Kennedy’s group at Harvard Medical School, the OPGs have been described as playing an important role in osmoprotection (Kennedy, 1982; Lacroix, 1989; Breedveld and Miller, 1994; Cayley et al., 2000; Bontemps-Gallo et al., 2017), in envelope structure (Delcour et al., 1992; Banta et al., 1998; Bontemps-Gallo et al., 2017), in virulence (Bhagwat et al., 2009) as well as in cell signaling (Fiedler and Rotering, 1988; Ebel et al., 1997; Bouchart et al., 2010). Among the different models used to study the biological function of this carbohydrate, *D. dadantii* is the most developed model for understanding their role in virulence and cell signaling.

The mutant devoid of OPG is described as having a complex pleiotropic phenotype: increased mucoid appearance (Breedveld and Miller, 1994; Ebel et al., 1997; Page et al., 2001), a decrease in motility (Fiedler and Rotering, 1988; Page et al., 2001; Bhagwat et al., 2009), and a loss of virulence (Bontemps-Gallo and Lacroix, 2015). The mucoid appearance of bacterial colonies is the consequence of activation of the RcsCDB two-component system (Bouchart et al., 2010; Bontemps-Gallo et al., 2013). This activation leads to up-regulation of the *eps* operon (Ebel et al., 1997; Bouchart et al., 2010), the genes of which are responsible for the synthesis of exopolysaccharides. The dramatic decrease in motility is also demonstrated to be a consequence of inactivation of the RcsCDB two-component system (Bouchart et al., 2010; Bontemps-Gallo et al., 2013; Bontemps-Gallo and Lacroix, 2015). Here, we showed that if EnvZ-OmpR is involved in co-regulation of motility, inactivation of the system cannot restore motility in a strain lacking OPGs (Figure 3).

Loss of virulence, certainly the most investigated phenotype, is more complex to explain. Several mutations have now been described that partially [in genes encoding RcsCDB (Bouchart et al., 2010), KdgR, PecT (Bontemps-Gallo et al., 2014)] or fully restore virulence [in the gene encoding PecS (Bontemps-Gallo et al., 2014)] in *D. dadantii*. Restoration of virulence in potato tubers, the reserve organs, depends only on the ability to restore full production of pectinase (Bontemps-Gallo et al., 2014). Restoration of virulence in non-reserve organs requires restoration of more factors, as bacteria will encounter several plant defense mechanisms (e.g., the oxidative burst) (Reverchon and Nasser, 2013; Bontemps-Gallo et al., 2014). In this study, we showed that inactivation of the EnvZ-OmpR system partially restores virulence in potato tubers (Figure 4) but not in chicory leaves (Figure 5). The result matched with the restoration of the pectinase production (Figure 1).

Finally, the second major finding of this study is the requirement for OPGs for the activation of the EnvZ-OmpR system. In *E. coli*, the EnvZ-OmpR system senses osmolarity in an unknown manner and modulates the expression of genes necessary for adaptation to the new conditions (Forst and Roberts, 1994; Castillo-Keller et al., 2006). This system is characterized both as a repressor (high osmolarity) and as an activator (low osmolarity) of *ompF* in *E. coli* (Lan and Igo, 1998). Surprisingly, in *D. dadantii*, the EnvZ-OmpR system only acts as an activator (Figure 6). This activation required OPGs in the periplasm (Figure 6). In contrast to RcsCDB (Bontemps-Gallo et al., 2013), periplasmic OPG concentration does not affect the level of activation of the EnvZ-OmpR system (Figure 8). The relationship between EnvZ-OmpR and OPGs is most likely indirect yet specific, since the CpxAR system was not affected by OPGs (Figure 6).

Several questions remain and require further investigations. Do other two-component systems need OPGs to be functional in *D. dadantii*? Preliminary data from our laboratory suggests that, among the 32 two-component systems, only RcsCDB and EnvZ-OmpR activation is affected by OPG presence/concentration. Does the specific relationship between the RcsCDB or EnvZ-OmpR system and OPGs also exist in phylogenetically closely related bacterial species? In non-pathogenic *E. coli*, inactivation of RcsCDB or EnvZ-OmpR restores motility in an *opgG* mutant (Fiedler and Rotering, 1988; Girgis et al., 2007). In *Salmonella enterica* serovar Typhimurium, inactivation of RcsCDB restores motility but not virulence in mice (Kannan et al., 2009). However, the relationship between OPGs and two-component systems has not been investigated in other bacteria. Finally, the more intriguing feature is the mechanism(s) by which OPGs modulate two-component system activation.

## Author Contributions

SB-G and J-ML conceived and designed the study, and wrote the manuscript. MC and SB-G performed all experiments with the assistance of EM, PG, and BD. MC, SB-G, EM, and J-ML analyzed the data.

## Conflict of Interest Statement

The authors declare that the research was conducted in the absence of any commercial or financial relationships that could be construed as a potential conflict of interest.
