# Supplementary material for: The EnvZ-OmpR Two-Component Signaling System Is Inactivated in a Mutant Devoid of Osmoregulated Periplasmic Glucans in Dickeya dadantii
Source: Front Microbiol. 2018 Oct 30;9:2459. doi: 10.3389/fmicb.2018.02459 (PMC6218677; doi:10.3389/fmicb.2018.02459)
Supplement: Supplementary file 1 [file Data_Sheet_1.DOCX]

Supplementary Material

**The EnvZ-OmpR Two-Component Signaling System Is Inactivated In A Mutant Devoid Of Osmoregulated Periplasmic Glucans In *Dickeya dadantii***

Caby Marine^1^, Sébastien Bontemps-Gallo^1‡^, Peggy Gruau^1^, Brigitte Delrue^2^, Edwige Madec^1^, Jean-Marie Lacroix^1^

*** Correspondence:** Corresponding Author: [jean-marie.lacroix@univ-lille1.fr](mailto:jean-marie.lacroix@univ-lille1.fr)

**Supplementary Figure 1.** Representative results for the phenotypic analysis of wild-type, *opgG*, *envZ*, *envZ opgG*, *ompR*, *ompR opgG* strains. (A) Pectate-lyase, (B) cellulase, (C) protease activities and (D) motility. Exoenzyme activities and motility were estimated on plates by measurement of the halo diameter. Bacteria (10^7^) were spotted onto protease, cellulase, protease plate or motility plate and revealed after 48h of incubation at 30°C. (E) Pathogenicity on potato tubers. Bacteria (10^7^) were inoculated into holes on potato tubers and incubated for 72h at 30°C.
