# Supplementary figures and images for: The EnvZ-OmpR Two-Component Signaling System Is Inactivated in a Mutant Devoid of Osmoregulated Periplasmic Glucans in Dickeya dadantii
Source: Front Microbiol. 2018 Oct 30;9:2459. doi: 10.3389/fmicb.2018.02459 (PMC6218677; doi:10.3389/fmicb.2018.02459)

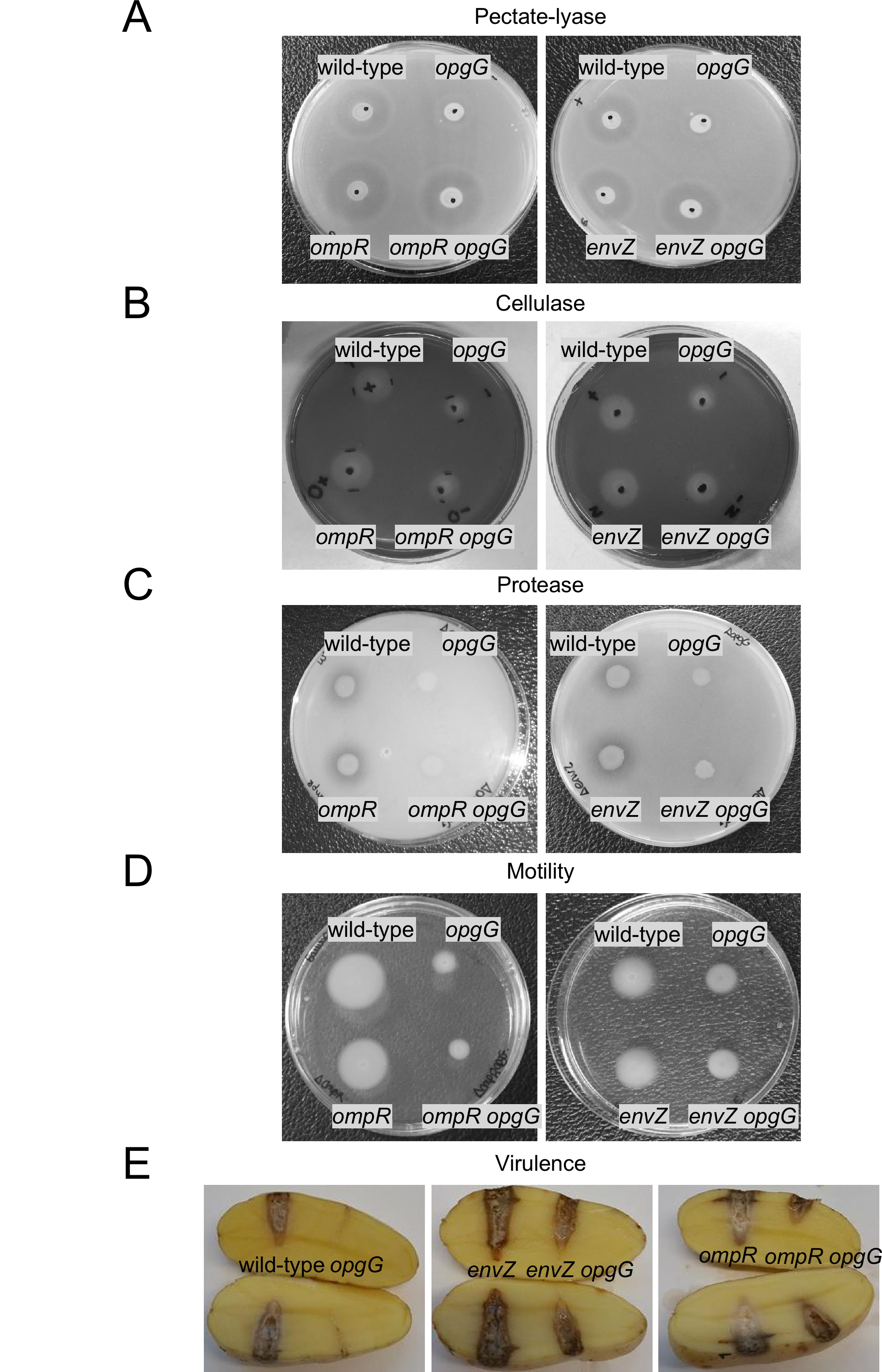

Supplement: Supplementary file 2 [file Image_1.TIF]
